# Supplementary material for: Rapid, high-throughput phenotypic profiling of endosymbiotic dinoflagellates (Symbiodiniaceae) using benchtop flow cytometry
Source: PLoS One. 2023 Sep 14;18(9):e0290649. doi: 10.1371/journal.pone.0290649 (PMC10501577; doi:10.1371/journal.pone.0290649)
Supplement: Attachment — Submitted filename: PLOSONE_ReviewResponse.pdf [file pone.0290649.s002.pdf]

Colin J. Anthony
Marine Laboratory, University of Guam
UoG Station, Mangilao, Guam, 96923

June 22, 2023

Dear PLOS ONE editor and reviewers,

Both reviewers acknowledged the scientific value of our research manuscript. All comments have been responded to, and we believe the manuscript is now fit for publication.

To address the largest concerns, we have reframed our manuscript to present the protocol as a method to quantify single-cell phenotypes and phenotypic variation of an assemblage. This interpretation relies less on fluorescence identifications, and instead takes advantage of flow cytometry's ability to detect small changes in cell phenotypes informed by a combination of red fluorescence, green fluorescence, forward scatter, and side scatter. Additionally, you will find that in this revision, we provide extensive additional references to the relevant literature to support our framework and interpretations.

In addition to this revised framework (reflected by the updated title), we have made modifications to figures and increased the robustness of our conclusions through more thorough use and discussion of statistical methodologies.

Below you will find specific responses to each concern. Reviewer responses are in normal font, while our responses to the reviewers are in ***bold and italic font***.

We are deeply appreciative of the reviewer's suggestions, and look forward to hearing from you.

Best Wishes,

Colin Anthony

—---

PONE-D-22-33653
High-throughput physiological profiling of endosymbiotic dinoflagellates (Symbiodiniaceae) using flow cytometry
PLOS ONE

In order to fit the new phenotypic framework, the title has been changed to: "Rapid, high-throughput phenotypic profiling of endosymbiotic dinoflagellates (Symbiodiniaceae) using benchtop flow cytometry".

Dear Dr. Anthony,

Thank you for submitting your manuscript to PLOS ONE. After careful consideration, we feel that it has merit but does not fully meet PLOS ONE's publication criteria as it currently stands. Therefore, we invite you to submit a revised version of the manuscript that addresses the points raised during the review process.

==============================
Thank you for your patience with your submission. One thing to really pay attention to with your resubmission is making sure that you have accurately cited the literature. For example, you are missing the Rosental et al, 2017 paper which first showed the utility of flow cytometery and non-stain assays on the coral and the symbiont cells. Additionally, reviewers brought up that you need to make sure that you do not "oversell" your work. While there are novel aspects, this is building on previous work, and it is important to acknowledge that. Also without other methods to confirm what you have found you must be careful with to not overstate your results.

We have used a citation management system to ensure that our citations are all present and properly formatted to PLOS ONE's preferences. We have also reframed our paper to discuss the characterization of phenotypes (instead of physiology). This is not only more conservative, but also more robust. We hope you find the revised manuscript satisfactory.

We look forward to seeing your resubmission.

Please ensure that your decision is justified on PLOS ONE's publication criteria and not, for example, on novelty or perceived impact.

For Lab, Study and Registered Report Protocols: These article types are not expected to include results but may include pilot data.
==============================

Please submit your revised manuscript by Apr 17 2023 11:59PM. If you will need more time than this to complete your revisions, please reply to this message or contact the journal office at plosone@plos.org. When you're ready to submit your revision, log on to https://www.editorialmanager.com/pone/ and select the 'Submissions Needing Revision' folder to locate your manuscript file.

Please include the following items when submitting your revised manuscript:

- A rebuttal letter that responds to each point raised by the academic editor and reviewer(s). You should upload this letter as a separate file labeled 'Response to Reviewers'.

- A marked-up copy of your manuscript that highlights changes made to the original version. You should upload this as a separate file labeled 'Revised Manuscript with Track Changes'.
- An unmarked version of your revised paper without tracked changes. You should upload this as a separate file labeled 'Manuscript'.

If you would like to make changes to your financial disclosure, please include your updated statement in your cover letter. Guidelines for resubmitting your figure files are available below the reviewer comments at the end of this letter.

If applicable, we recommend that you deposit your laboratory protocols in protocols.io to enhance the reproducibility of your results. Protocols.io assigns your protocol its own identifier (DOI) so that it can be cited independently in the future. For instructions see: https://journals.plos.org/plosone/s/submission-guidelines#loc-laboratory-protocols. Additionally, PLOS ONE offers an option for publishing peer-reviewed Lab Protocol articles, which describe protocols hosted on protocols.io. Read more information on sharing protocols at https://plos.org/protocols?utm_medium=editorial-email&utm_source=authorletters&utm_campaign=protocols.

Our manuscript is accompanied by an in-depth lab protocol on protocols.io.

We look forward to receiving your revised manuscript.
Kind regards,
Nikki Traylor-Knowles, Ph.D.
Academic Editor
PLOS ONE
Journal Requirements:
When submitting your revision, we need you to address these additional requirements.
1. Please ensure that your manuscript meets PLOS ONE's style requirements, including those for file naming. The PLOS ONE style templates can be found at
https://journals.plos.org/plosone/s/file?id=wjVg/PLOSOne_formatting_sample_main_body.pdf
and
https://journals.plos.org/plosone/s/file?id=ba62/PLOSOne_formatting_sample_title_authors_affiliations.pdf
2."Please update your submission to use the PLOS LaTeX template. The template and more information on our requirements for LaTeX submissions can be found at http://journals.plos.org/plosone/s/latex."
3. Thank you for stating the following in the Acknowledgments Section of your manuscript:

"We would like to thank Rebecca Salas for running the upside-down jellyfish acclimation experiment. We would also like to thank MacKenzie Heagy for illustrating the coral dinoflagellate assemblages shown in Fig 5. Guam NSF EPSCoR directly supported this work through the National Science Foundation award OIA-1946352. Any opinions, findings, conclusions, or recommendations expressed in this contribution are those of the authors and do
not necessarily reflect the views of the National Science Foundation"
We note that you have provided funding information that is not currently declared in your Funding Statement. However, funding information should not appear in the Acknowledgments section or other areas of your manuscript. We will only publish funding information present in the Funding Statement section of the online submission form.

Please remove any funding-related text from the manuscript and let us know how you would like to update your Funding Statement. Currently, your Funding Statement reads as follows:
"CJA, CL, and BB are all directly supported by NSF Guam EPSCoR (https://guamepscor.uog.edu/) through the National Science Foundation (https://www.nsf.gov/) award OIA-1946352. The funders had and will not have a role in study design, data collection and analysis, decision to publish, or preparation of the manuscript"

The funding information has been removed from the acknowledgements. You may maintain the funding statement as it stands with slight modification. The last sentence should read as follows: "The funders did not and will not have any role in the study design, data collection, data analysis, decision to publish, nor preparation of the manuscript."

Please include your amended statements within your cover letter; we will change the online submission form on your behalf

[Note: HTML markup is below. Please do not edit.]

Reviewers' comments:

Reviewer's Responses to Questions
Comments to the Author

1. Does the manuscript report a protocol which is of utility to the research community and adds value to the published literature?
Reviewer #1: Yes
Reviewer #2: Yes

We are glad both reviewers see the value in our work. We thank both of them for their input. Both reviewers were exceptionally insightful, and we hope they find the revised manuscript suitable for publication.

---

2. Has the protocol been described in sufficient detail?

To answer this question, please click the link to protocols.io in the Materials and Methods section of the manuscript (if a link has been provided) or consult the step-by-step protocol in the Supporting Information files.

The step-by-step protocol should contain sufficient detail for another researcher to be able to reproduce all experiments and analyses.
Reviewer #1: No
Reviewer #2: Yes

Based on reviewer #1's suggestions, we added additional details to the manuscript, protocol, and github repository. The narrative has been streamlined, small things (e.g. pre-load vortexing and within-machine mixing) have been made more conspicuous or added to both the manuscript and protocol as necessary. All code and required databases to duplicate analyses have been deposited on GitHub. The procedure is fully outlined in protocols.io and adequately referenced in the manuscript when needed.

Now that we have responded to and resolved every comment by both reviewers, we hope you will agree that our methods are presented in a comprehensive and replicable fashion.

---

3. Does the protocol describe a validated method?

The manuscript must demonstrate that the protocol achieves its intended purpose: either by containing appropriate validation data, or referencing at least one original research article in which the protocol was used to generate data.
Reviewer #1: No
Reviewer #2: Yes

The manuscripts presenting similar concepts on which our method is built are as follows: Apprill et al. 2007, Lee et al. 2012, and Munoz et al. 2018. We used these concepts presented in the literature to develop our protocol, while additionally fine tuning the definitions and framework around our quantified metrics. Apprill et al. 2007 served as the basis to the idea of phenotyping. In this revision, you will find that we reframed the paper to discuss phenotyping based on four widely accepted flow cytometric parameters (red fluorescence, green fluorescence, forward scatter, and side scatter). This phenotypic framing is empirically backed, more valuable, and more robust than our original framework where we focused too heavily on photopigment abundance and identification (as pointed out by reviewer #1).

Other research articles referenced in the introduction do not use our exact protocol, but our protocol builds directly on this literature (Olsen et al. 1983; Dubelaar and Jonker 2000; Becker et al. 2002; Lee et al. 2012; Chen et al. 2017). Within this literature flow cytometry has been validated multiple times as a reliable method for quantifying photopigments (Becker et al. 2002; Freitas et al. 2014; Chen et al. 2017), each using similar laser/filter profiles.

Our paper also uses an original experiment with the model organism Cassiopea as a protocol demonstration to discuss the analysis, interpretation, and visualization. We also present two optimization frameworks with original data to intimately discuss sample preparation and degradation. We hope the reviewers are satisfied with this extensive and thorough revision highlighting the robust literature behind our foundational concepts.

---

4. If the manuscript contains new data, have the authors made this data fully available?

The PLOS Data policy requires authors to make all data underlying the findings described in their manuscript fully available without restriction, with rare exception (please refer to the Data Availability Statement in the manuscript PDF file). The data should be provided as part of the manuscript or its supporting information, or deposited to a public repository. For example, in addition to summary statistics, the data points behind means, medians and variance measures should be available. If there are restrictions on publicly sharing data—e.g. participant privacy or use of data from a third party—those must be specified.
Reviewer #1: Yes
Reviewer #2: Yes

Although it was not requested, we have deposited code and databases into a public GitHub repository in addition to the originally included protocols.io and supplemental files.

---

5. Is the article presented in an intelligible fashion and written in standard English?

PLOS ONE does not copyedit accepted manuscripts, so the language in submitted articles must be clear, correct, and unambiguous. Any typographical or grammatical errors should be corrected at revision, so please highlight any specific errors that need correcting in the box below.
Reviewer #1: Yes
Reviewer #2: Yes

---

6. Review Comments to the Author

Please use the space provided to explain your answers to the questions above. You may also include additional comments for the author, including concerns about dual publication, research ethics, or publication ethics. (Please upload your review as an attachment if it exceeds 20,000 characters)

Reviewer #1: The authors present some groundwork in developing new protocol points and a new application of flow cytometry in understanding the photosynthetic response of Symbiodiniaceae. As someone who has worked on developing new technologies and uses for flow cytometry I found the premise very interesting. However, ultimately he paper fell short, and/or the authors over-reached, in presenting the data generated.

Generally, the language has been modified to present our protocol more conservatively. More specifically, the paper has been reframed to present flow cytometry as a way to phenotype Symbiodiniaceae cells, a currently difficult to access level of data resolution. We take a step back and discuss fluorescence less specifically, instead we use it as a representation of the underlying phenotype. You will also find that now our claims are supported by more extensive references to the relevant literature. We have included substantial background on flow cytometry, thus validating the methodology by reference to other relevant work in the field. We thoroughly appreciate your insight from the initial review, and as you read through the revised version, we hope you find the revised framework robust, convincing, and valuable.

My main issues concern the following (1) for a new method which makes big statements about its use in photo physiology, there is no ground truthing (i.e., comparative HPLC or even simple chl extractions),

Thank you for the suggestion. We would like to point out that while this is a lesser used application of flow cytometry, this is not a new method. Flow cytometry has been used to quantify photopigments since the early 80s (Olsen et al. 1983; Dubelaar and Jonker 2000; Becker et al. 2002; Apprill et al. 2007; Lee et al. 2012; Chen et al. 2017). Flow cytometry has been validated against HPLC and other methods (e.g. Becker et al. 2002; Freitas et al. 2014). A thorough background on flow cytometry uses has been included in the introduction, and from there, you will find a proper acknowledgement of this literature throughout the methods, results, and discussion. We acknowledge that we over-sold the novelty of our protocol, and we also acknowledge that we didn't quite understand the importance of our protocol until we received your thorough and insightful review. We have reframed our manuscript to accentuate the fact that there is not a lack of methodological validation, but instead a lack of an in-depth, replicable protocol with proper consideration of Symbiodiniaceae phenotypes. This is accompanied by a focused discussion of the value and potential future applications of our method to resolve issues in coral biology.

(2) this is extra problematic for flow cytometry where there is inability to translate RFUs to biologically meaningful units or understand effect sizes,

We understand your perspective. Our method is not intended to provide absolute measures of pigment quantities but to facilitate comparisons of, for example, treatment groups. The method presented by us provides measures or relative change, similar to other applications such as

qPCR for relative comparisons of gene expression. The expanded literature review on flow cytometry and its applications in quantifying photopigments should further clarify this issue. That said, we revised the framework to interpret fluorescence as a representation of the cell's underlying phenotype. This concept acknowledges the possible ambiguity of distinguishing individual pigments using fluorescence, but links differentiation in fluorescence to differences in the cell's phenotype. Such differentiation is reasonably interpreted as caused by changes in the abundance of LHC-associated photopigments (as discussed in much of the current literature). However, localization of chloroplasts or even cell size may impact fluorescence signatures. Our revised focus on the phenotype is not impacted by such possible differences in the underlying causes of fluorescence variation. Our Cassiopea experiment serves as a model for discussing the analysis and interpretation of the data generated by our method, for example by presenting levels of data resolution (discussed later in this review response). Thank you very much for your critical feedback which helped us in rethinking our framework.

(3) there is almost no acknowledgement of the potential issues with this protocol/interpretation – e.g., fluorescence spill over, the sensitivity of flow cytometers to gain settings. In the face of these issues, the very specific and detailed statements that the authors make to relate them to photophysiology are not well enough supported. I think the paper would need major revisions to reflect what are interesting hypotheses vs. proof of concept.

Thank you for the suggestion. We have added discussion of limitations to our approach and interpretation throughout the manuscript. To address spillover, we added multiple sentences discussing spillover/spectral overlap potential, a characteristic visualized in Figure 1. To address sensitivity of flow cytometers to gain settings, we have integrated more conspicuous comments into the protocol file (File S1/protocols.io) as well as a comment on it in section 1.4.2. Overall, we address the reviewers' concerns in our discussion where we acknowledge potential biases and limitations with the method. Topics of discussion now include the acknowledgement of fluorescence spillover/spectral overlap, flow cytometric sensitivity, and consistent gain settings. (Protocol, Methods, Discussion).

One thing that we made more obvious within the protocol is our use of fluorescence beads and compensation testing to check for machine consistency. As flow cytometers are used, they may require calibration. This is easily checked with fluorescent beads. A process that is now implemented into the protocols.io (e.g. step 4). That said, it was not an issue for our machine as we did no cross machine analyses, nor was any of our data generated across vast time scales that may lead to a difference in the flow cytometer's sensitivity. Our gain settings are also always set exactly the same, as we have designed the workflow to load automatically with identical settings. These points are mentioned in the protocols.io. 2 out of 3 assays presented here were processed within a single run. The third essay required 6 runs processed within 24 hours. Therefore, it was not necessary to check gain settings with fluorescent beads. The purposeful design of experiments to be processed within a single plate (48 wells) also increases confidence

in our interpretation of effect sizes by removing the potential for batch effects. We have used our cytometer for 2 years now, and fluorescent bead readings have not changed but instrument validation at regular intervals is important for users of flow cytometry to ensure consistency of results. (Comments addressing potential pitfalls in using flow cytometry have been added to the protocol file (File S1/protocols.io).

Thank you for the comment. The newly integrated discussion points and explanations should resolve this issue. We appreciate your insight. It has improved the quality of the paper.

In a lesser issue, the paper has a mixed identity of two very distinct projects that are never tied together well: a methodological step in preservation and cell counting, and a deeper dive into autofluorescence and functional profiling.

You may find that the discussion is much better tied together now as the Cassiopea experiment is now included in the "Protocol performance and optimization section". This helps the reader realize that the Cassiopea experiment is a similar demonstration of best practice and performance. Specifically, we now use it to demonstrate the method's level of resolution (thousands of cells vs a handful of hosts). Each level of data resolution is valuable, and should be considered. This is well integrated into all sections discussing Cassiopea, but is best addressed in paragraph three of section 4.2.

The first aspect, methodology, is relatively straight forward. I appreciate having some parameters for best practices with pigment analysis and cells counting. One thing the authors should consider though is that cells settle during processing, and while waiting to be processed. So best practices are normally to vortex the sample right before loading into the flow cytometer so that the sample is homogenized and the counts are accurate for doing a concentration by run time * flow rate.

This was considered. As described in the protocol, cells are vortexed at every stage of preparation to maintain slurry homogeny. Additionally, samples are vortexed prior to being loaded into the cytometer. Additionally, samples are mixed for 7 seconds by the flow cytometer before counting cells. All of this information is in the protocols.io file (steps 5.5, 5.6, 7, 7.2, 7.3, 8.5). Mentions have been made more conspicuous in the protocols document. We also added a brief reference to this in the manuscript (e.g. section 4.2, paragraph 2). Thank you for the comment, we hope the more conspicuous language is suitable for the resolution of your concern.

Doing the whole sample is also fine but you run into issues, as did the authors, with the clumping of settled cells and variable counts for samples coming later in the processing. Please consider this in the discussion.

Clumping only occurred at high cellular concentrations and was not due to settling. We presume this is caused by an extremely high concentration of cells. This limits effective vortexing by the

machine. This is why we recommend a 10 fold dilution vs an undiluted sample because clumping is not an issue at more diluted concentration, but there is still sufficient sampling, as indicated by Figure 3. The point of that assay was to demonstrate the importance of a well-calibrated protocol and what an ideal cellular concentration looks like. These points have been made more explicit in the revised version.

Again, thank you for mentioning this. We did not make these aspects of the method conspicuous enough. These points were present in the discussion and were the primary reason for presenting Figure 3. In order to resolve these criticisms, we have stated these 'best practice' techniques more explicitly in section 4.2.

The second aspect, empirical application, has several issues, that unfortunately make it unsuitable for publication. Some of these were stated above. Firstly, from my experience, there is quite a lot of spectral overlap of pigments across channels, but no clear discussion of this or how compensation was done.

These are naturally difficult things that come with working with cellular autofluorescence. Typically autofluorescence can be compensated for with the use of artificial fluorophores. However, we are only detecting autofluorescence. Additionally, the profiles we discuss as highly relevant (GRN-B, RED-B) are not affected by compensation of other lasers (we checked). That said, any discussion of the RED-R value or the YEL-B value has been removed from the manuscript. The original attempt to include them was not robust. Now, in our new framework we only include green fluorescence, red fluorescence, forward scatter, and side scatter; these are the most widely used metrics in flow cytometry. That said, we have included a substantial discussion of spectral overlap and autofluorescence. This is not only visible in figure 1, but you will find that it is now thoroughly discussed throughout the manuscript, primarily in sections 2.2 and 4.1. Thanks to your suggestion, the revision discusses all of these issues throughout the manuscript. We are confident that our protocol follows best practice and you may now find it more explicitly stated that we have thoroughly considered possible sources of bias, and each step has been optimized or considered accordingly.

The analysis of the data with respect to the jelly experiment is unclear. There is no indication of how the 4 samples were dealt with in the analysis. Is Fig. 4 one sample from one jelly in each treatment? Samples from multiple jellies plotted together? How was the gain accounted for? Did the analysis take into account that the experimental replication was 4 not 20,000? The differences between treatments seem so small even on a log scale against RFUs and the extremely low p-values, despite little visible grouping suggests that this is being driven by cell numbers rather than experimental treatments. If you randomly sampled 100 cells, is there still a difference?

We agree with this comment and have resolved the issues as follows. In addition to the revisions to the manuscript (Lines 148-174), we have deposited all data and code into a github to make

sure the data analysis and graphing are replicable. Fig 4 was all jellies plotted together; however, we have used your comment as the basis to rework the role of Figure 4 and our acclimation experiment. We now use it as a representation of understanding the underlying data structure. It is now represented by a PCA, summarized to each jellyfish host, illustrating the phenotypic partitioning of Cassiopea after exposure to different light conditions. We then included 8 graphs to visualize all 4 phenotypic parameters and 2 levels resolution. Level one (Figure 4B-E) represents distributions built from individual Symbiodiniaceae cells independent of the host jellyfish (n=16,134, 18,464, 19,821). Level 2 (Figure 4F-I) represents distributions built from the mean phenotypes for each jellyfish (n = 4,4,5). We now use this to discuss data resolution and effect size, where we recommend considering a combination of the entire assemblage's phenotypic variation in addition to the average phenotype within each jellyfish. Both are valuable, which we discuss in section 4.2, paragraph 3.

In reference to gain, gain is not a factor for the included experiments because all samples were processed on the same plate, so all samples were processed with the exact same settings. However, if a researcher would like to use data from multiple cytometry runs, how to handle gain is discussed in the protocols.io and referenced in the manuscript.

Subsampling of the data is an interesting suggestion. We tested subsampled distributions out of curiosity and the statistical conclusions did not change. We tested the strength of the signal by randomly sampling 90%, 75%, 50%, 25%, 10%, and 1% of the cellular observations to see if the conclusion changes when analyzing data at the scale at the cellular level. As anticipated, the data structure was always maintained, but the statistical power behind the data weakened before totally breaking down between 10% and 1% data subsamples. That said, we felt this analysis was tangential and not necessary to the manuscript. Instead, we maintained our original analysis and added a section about the host. We decided to use this as an opportunity to discuss the protocol's ability to be able to detect even minute variation in a Symbiodiniaceae population due to extremely high biological replication (cells) within the machine. The host is simply an added random factor to increase the strength of our cellular replication.

Thank you for the comment. These were all valid and insightful concerns, and we appreciate the comment. We believe the restructuring of the analysis, figure 4, and transparent discussion has resolved this issue.

Can the authors include some of the plots used for gating forward and sidescatter, particularly those used to "remove noise".

We have modified Figure 1 to include an example plot used to select Symbiodiniaceae based on RED-B. Other examples are readily available throughout the protocols.io document (File S1).

The results described in lines 196-203 are difficult to parse. Statistics could be included where the trends are mentioned rather than before.

This has been rewritten to be more consumable to the reader. Thank you for the comment.

In the fig 4 caption, the authors acknowledge that the effect size is small but the following discussion misses this point entirely.

The 'small effect size' should be less of an issue now with the revised statistical resolution and added discussion paragraph. Thank you for the concern. We find the new structure much more transparent and convincing.

The discussion is just largely overinterpreted with no acknowledgements of the major issues I mentioned above. Even though I enjoyed reading it in parts and thinking about these hypotheses in terms of shifts in pigments that might happen, the data are just not there to support it given the lack of groundtruthing, the small effect size, and the inability to make biological sense of RFU's.

We appreciate this comment. You will now find that in addition to building up the methodological background in the Introduction, we have reframed the paper to no longer focus on specific pigments. Instead focus on how they represent phenotypes. Any discussion pertaining to "biological sense of RFU's" is presented both heavily backed by previously published literature and presented very conservatively. We hope you find this satisfactory.

The end of the discussion, line 281. This paragraph is missing some context. There are papers that use flow cytometry to identify genetic groups and look at nutrients (McIlroy et al. 2020), it's missing some cool flow cytometry work on functional groups across depth from Apprill et al. 2007 Coral Reefs; there is some great cell cycle work recently in tivey et al. 2020. There is more to functional diversity then pigment content.

This comment was the center of our revision. Specifically Apprill et al. 2007 allowed us to reframe our methodology to broad quantification of phenotypes. The discussion has been largely restructured to focus on phenotyping and overall made more conservative to acknowledge the most empirically supported concepts. The suggested literature has been integrated primarily into the introduction, but you may also find this literature (and more) referenced periodically throughout the entirety of the manuscript. All of your previous and current suggestions have been fully integrated. We trust you will find our edits satisfactory.

Fig. 5 - Are these PCA's real or theoretical? If theoretical why not plot the high and low light groups generated from this. Please be explicit about this.

These PCAs are theoretical, as indicated in the figure legend. We simply used them to illustrate a theoretical application that addresses a research area we believe fruitful exploring further. You may now find that this has been made more obvious and better integrated into the overall narrative. The figure is not directly related to our example application with light groups. That said, we added a PCA to the Cassiopea assay to improve the conceptual connection between figure 4 and figure 5. Figure 5 has also been modified to include the four phenotypic traits maintained in this work (green fluorescence, red fluorescence, forward scatter, and side scatter). The restructured discussion should make this more obvious in addition to modifications to the figure caption. Specifically, the end of the figure caption now reads as follows, "As indicated by arrows, the combination of genetic diversity and phenotypic profiles has the potential to allow for the classification of Symbiodiniaceae assemblage functional strategies into homogenous, plastic, redundant, or diverse categories upon implementation into other relevant workflows. PCA datasets are theoretical and were generated and analyzed using R."

Reviewer #2: In the manuscript entitled: "High-throughput physiological profiling of endosymbiotic
dinoflagellates (Symbiodiniaceae) using flow cytometry" Anthony et al, describe a new way of flow cytometry analysis of the symbiont algae in corals. What is unique in this work is while flow cytometry was used in the past for the algae analysis, they take the fluorescent properties to analyze the state on a single cell level, which flow cytometer is enabling. Interestingly they are doing it without adding too much additional tools, making this method ready to be applied in many labs around the world.
This is a well written manuscript with a good and applicable idea, which can be used in many laboratories working on algae in different organisms and not only corals.

We are glad you like it, and we hope to see its future integration and development. We regularly use it in our lab, and find it extremely informative.

This reviewer has only few minor comments:

Figure 5 - Labels of 'Low complexity' and 'High complexity' are both too close to the branching coral diagram and should moved to avoid confusion. I would suggest moving each label underneath its relevant diagram.

Great suggestion. The labels have been moved to avoid confusion. (Fig 5). We also modified the figure to now better reflect our final parameters included in the phenotypic profile: red fluorescence, green fluorescence, forward scatter, and side scatter.

Methods - Line 82 - comma needed after "laser excitation".

This has been corrected. Thank you.

Methods - Line 102 - it is not clear how the authors prepared the plate.
" three across-well and two within-well replicates were used for data collection"
What does that mean? How many wells were used and how many replicates?

Three wells were used per replicate, and each well was sampled twice by the flow cytometer. Thank you for this. The statement has been clarified in the methods section.

General Question: Please mention how you kept the experimental animals? Like a 'Animal Husbandry' section in the methods?

An animal husbandry/culture explanation has been added to the methods section. Thank you for mentioning this.

Methods - Line 103 - 111 (Assay 2) - Initial cell concentration should be noted in the methods.
(It appears as a note in the supplementary protocol File S1)
(It also appears on line 168)

Starting cell concentration has been integrated into the methods. Thank you for the suggestion.

General Question about Figure 2: In the text the authors highlighted that certain conditions bring the most consistent results which were indicated by the dashed boxes. Please indicate how conclusions are determined.

Thank you for the comment. This is important information that we missed. We added our selection criteria to the materials and methods section, which was the use of pairwise Dunn's tests to infer statistical groupings. We also modified the figure to include statistical groupings that are indicated by letters. This provides more context to our final 2 hour suggested cutoff. We appreciate the comment.

---

7. PLOS authors have the option to publish the peer review history of their article (what does this mean?). If published, this will include your full peer review and any attached files.

If you choose "no", your identity will remain anonymous but your review may still be made public.

Do you want your identity to be public for this peer review? For information about this choice, including consent withdrawal, please see our Privacy Policy.
Reviewer #1: No
Reviewer #2: No

[NOTE: If reviewer comments were submitted as an attachment file, they will be attached to this email and accessible via the submission site. Please log into your account, locate the manuscript record, and check for the action link "View Attachments". If this link does not appear, there are no attachment files.]

While revising your submission, please upload your figure files to the Preflight Analysis and Conversion Engine (PACE) digital diagnostic tool, https://pacev2.apexcovantage.com/. PACE helps ensure that figures meet PLOS requirements. To use PACE, you must first register as a user. Registration is free. Then, login and navigate to the UPLOAD tab, where you will find detailed instructions on how to use the tool. If you encounter any issues or have any questions when using PACE, please email PLOS at figures@plos.org. Please note that Supporting Information files do not need this step.

In compliance with data protection regulations, you may request that we remove your personal registration details at any time. (Remove my information/details). Please contact the publication office if you have any questions.
